# Uptake of intermittent preventive treatment for malaria during pregnancy with Sulphadoxine-Pyrimethamine in Malawi after adoption of updated World Health Organization policy: an analysis of demographic and health survey 2015–2016

**DOI:** 10.1186/s12889-020-08471-5

**Published:** 2020-03-16

**Authors:** Steven Chifundo Azizi

**Affiliations:** Malawi Defence Force, Malawi Military Health Services, Kamuzu Barracks, Lilongwe, Malawi

**Keywords:** Pregnancy, Intermittent preventive treatment, Sulphadoxine-pyrimethamine, Malaria, Antenatal care, Malawi

## Abstract

**Background:**

Malawi adopted the 2012 updated Word Health Organization (WHO) Intermittent preventive treatment of malaria during pregnancy with sulphadoxine-pyrimethamine (IPTp-SP) policy in 2013. This study aimed to estimate the proportion of and identify factors associated with the uptake of at least three doses of IPTp with SP among pregnant women in Malawi after the adoption and operationalisation of updated WHO IPTp-SP policy.

**Methods:**

The 2015–16 Malawi Demographic and Health Survey dataset was analysed. Of 1219 women aged 15–49 years who had live births and the children were born after the date of July 2015, 1069 women were included in the analysis. Bivariate and multiple logistic regression were used in data analysis. The statistical analysis took into account a complex survey sample design.

**Results:**

Of the 1069 women, 447 (42, 95% CI: 38.1–45.6) received three (optimal) or more doses of IPTp-SP. Less than half (47%) managed to attend at least four antenatal care (ANC) clinics. Only 52% received optimal SP doses among those who made at least four ANC visits. Only the number of ANC visits was associated with the optimal uptake of SP. Women who attended ANC three times only and those who visited ANC once or twice only were less likely to receive at least three doses of SP than those who managed to attend ANC at least four times during pregnancy (AOR = 0.71, 95% CI 0.49–1.02) and (AOR = 0.12, 95% CI 0.06–0.21) respectively.

**Conclusions:**

To achieve effective malaria prevention in pregnancy, IPTP-SP is used alongside other interventions. However, there is low uptake of optimal SP doses in Malawi, and this seems to be associated with the number of ANC visits. Moreover, there is limited effectiveness of an increased number of ANC visits on the uptake of optimal SP doses. Further research should be done to explore health systems factors affecting uptake of optimal IPTp with SP doses during pregnancy.

## Background

With an estimated 125 million pregnant women at risk of malaria infection each year globally, 30 million of them emanate from sub-Saharan Africa (SSA) [1]. Malaria in pregnancy (MiP) ranks among the major public health problems in SSA [2–5]. Available statistics have revealed that 75,000–200,000 infants and 10, 000 women deaths annually are attributed to MiP [1, 6]. Pregnant women are susceptible to severe *Plasmodium falciparum* infection because of alteration of acquired antimalarial immunity due to parasites (VAR2CSA) that sequester in the placenta [7, 8]. The *Plasmodium falciparum* impairs the capacity of the placenta to transport amino acids from maternal blood to the foetus, and therefore contributing to low birth weights (LBW) [9]. Other consequences of malaria infection in pregnancy are increased risk of severe anaemia, cerebral malaria, pre-term delivery, intrauterine growth retardation, maternal death and increased risk to unborn baby from miscarriage [10–13]. It has been estimated that *Plasmodium falciparum* infections in pregnancy contribute to around 11% of neonatal deaths due to LBW in endemic areas in Africa [6, 14, 15].

In Malawi, malaria is hyper-endemic and transmission trend rises in the rainy season (October to April) and in areas with high temperatures especially around lakeshore and lower Shire Valley [16]. Ninety-eight percent of malaria infections are caused by *Plasmodium falciparum* transmitted by *Anopheles funestus*, *A. gambiae*, and *A. arabiensis* mosquito vectors [16]. To mitigate malaria burden among pregnant women, Malawi was the first country to introduce Intermittent Preventive Treatment of malaria during pregnancy with sulphadoxine-pyrimethamine (IPTp-SP) in 1993 [17]. World Health Organization (WHO) recommends a package of three interventions for malaria prevention and control in pregnancy, which includes the administration of intermittent preventive treatment with sulfadoxine-pyrimethamine during pregnancy, use of insecticide-treated nets (ITNs), and prompt and effective treatment of malaria in pregnant women [18]. Sulphadoxine-pyrimethamine (SP) is an antifolate drug that inhibits cell multiplication of malaria parasites hence, placental malaria infection is prevented or *Plasmodium falciparum* active in the placenta is controlled [7].

In 2004, WHO recommended a minimum of two doses of IPTp with SP alongside other malaria prevention and control approaches during pregnancy [19, 20]. In October 2012, however, WHO updated the policy to at least three doses after acknowledgement from Evidence Review Group (ERG) that reviewed research evidence on the efficacy of IPTp-SP and its adverse outcomes in preventing MiP [21–26]. The updated policy further says that IPTp with SP should be administered at each antenatal visit, with the first dose to be given early in the second trimester and successive doses to be administered at monthly intervals until the time of delivery [27]. Malawi adopted the updated policy in 2013 [28]. WHO envisioned the policy would increase IPTp-SP uptake because the World Health Organisation also recommended at least four ANC visits during the second and third trimesters of pregnancy under the Focused antenatal care (FANC) model [21, 29]. Therefore there is ample chance to attain a high proportion of women receiving at least three doses; and if a pregnant woman does not receive SP at each scheduled ANC visits within the recommended IPTp-SP administration period, it would be deemed as a missed opportunity [20].

Since Malawi adopted and started implementing the updated WHO IPTp-SP policy, there is paucity of studies that nationally managed to estimate proportions of uptake of at least three doses of IPTp with SP and identified factors associated with it among pregnant women in Malawi such as Azizi et al. study [30]. Azizi et al. study, however, is limited to a particular district. On the other hand, the Malawi Demographic and Health Survey of 2015–2016 (MDHS) did not analyse data to specifically highlight the uptake of three or more doses of IPTp-SP after adoption and operationalisation of the 2012 updated WHO IPTp-SP policy. MDHS made a general estimate of uptake of three or more doses of SP without considering under what policy were the pregnant women receiving the SP during the gestation period. This estimate is reflected in a study by Nkoka et al. that used MDHS dataset [31]. Globally, studies have revealed that uptake of at least two doses of IPTp-SP is associated with number of ANC visits [5, 22, 30–39], directly observed therapy (DOT) [5, 30, 38, 40, 41], residential area [22, 30, 42], age of woman [42], education level and socioeconomic status [43], parity [5, 42], timing of initial ANC visit [5, 31, 44], knowledge about malaria/IPTp-SP [5, 38] and stockouts of the commodity [5, 38, 45]. This study aimed to contribute to identifying factors associated with completion of recommended doses of SP during pregnancy post-adoption of the updated WHO policy in resource-limited settings from a nationally representative sample. The aims of the study were to estimate the proportion of and identify factors associated with the uptake of at least three doses of IPTp with SP (IPTp3+) among pregnant women in Malawi after the adoption and operationalisation of the 2012 updated WHO IPTp-SP policy.

## Methods

### Study design and study setting

This was a cross-sectional survey carried out to provide estimates of basic demographic and health indicators for the Malawi nation. The study was nationally representative, with all 28 districts included in the sample, stratified by residential area (urban and rural). It yielded 56 sampling strata.

Weighted sample was selected in two stages. In the first stage, 850 standard enumeration areas (SEAs) also known as clusters (173 SEAs in urban areas and 677 in rural areas) were selected with probability proportional to the SEA size. The list of households in each selected SEA served as the sampling frame for the second stage. Some of the selected SEAs were large, hence to reduce the work of household listing, each large SEA (more than 250 households) was segmented. From each of the segmented SEAs, one segment was selected for the survey with probability proportional to the segment size. In the second stage, a fixed number of 30 households per urban cluster and 33 per rural cluster were randomly selected from each cluster’s household listing. A total of 27,516 households were selected, of which 26,564 were occupied. Among the occupied households, 26,361 were interviewed (99% response). From the interviewed households, 25,146 eligible women were identified for individual interviews and only 24,562 women were successfully interviewed (98% response). All women aged 15–49 years old were interviewed if they were permanent residents of, or had stayed in the household in the previous night before the survey in the period between October 2015 and February 2016. A detailed methodology has been published elsewhere [46].

### Study population

The target population was all Malawi resident women aged 15–49 years with live births one-year-old or less preceding the survey, between the years of 2015 and 2016. The study was based on data from the 2015–16 Malawi Demographic and Health Survey. Malawi National Statistical Office conducted the survey from 19 October 2015 to 18 February 2016.

### Inclusion and exclusion criteria

The analysis included all women age 15–49 with live children born at least in August 2015, because the implementation of the updated policy was nationally full-fledged in October 2014. On the other hand, study participants who had missing data on at least one of the key variables used in the analysis were excluded from the analysis.

### Sample selection

**Fig. 1:**
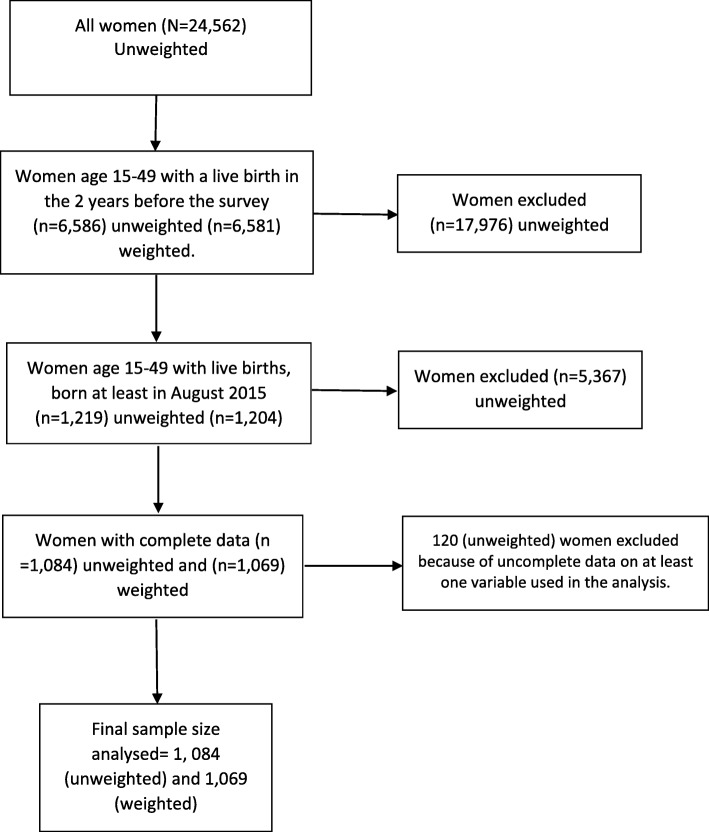
Flow Diagram of the included sample in the study

Out of 24, 562 women who completed the survey interviews, 6586 had live birth in the 2 years before the survey. Of the 6586 women, 1219 had children who were born after the month of July 2015. About 120 women were excluded from analysis because of lack of information on at least one variable used in the analysis. The final sample size used in the analysis was 1, 084 (unweighted) and 1069 (weighted) (Fig. 1).

### Data extraction and variables used in the analysis

Guided by literature review, data were extracted for analysis based on variables that were theoretically and empirically linked to uptake of IPTp-SP as follows: (a) *the outcome variable* was IPTp-SP uptake, categorised as two doses or less (≤2 doses) and three doses or more (3+ doses, also known as optimal dose); (b) *explanatory variables* were woman’s residential area, woman’s level of education, woman’s age, woman’s occupation, wealth, marital status, region, parity, timing of the first ANC visit, and number of ANC visits (Table 1). The data covering these variables were extracted from the 2015–16 MDHS women dataset. The data were collected using the woman’s questionnaire.

**Table 1:** Variables used in the study

Outcome variable	Definition	Category
IPTp-SP uptake	Two or less (≤2) doses is incomplete and three or more (3+) is optimal	≤2 doses
		3+ doses
**Explanatory variable**		
Residence	Area of woman’s residence (urban or rural)	Rural
		Urban
Education	Level of education of woman	No formal education
		Primary
		Secondary or higher
Age	Age group of a woman	15–19
		20–24
		25–29
		30–34
		35–39
		40+
Occupation	Woman’s occupation	Unemployed
		Self-employed
		Employed
Wealth	Household’s wealth from which a woman is an occupant	Poorest
		Poorer
		Middle
		Richer
		Richest
Marital status	Marital status of a woman	Married
		Divorced/separated/widowed
		Never married
Region		Southern
		Central
		Northern
Parity	Number of birth that a woman had after 20 weeks gestation	One child
		Two children
		Three children
		Four children
		Five or more children
Timing of 1st ANC visit	Age (in months) of the pregnancy a woman visited antenatal care clinic (ANC) for first time	1st trimester (1–12 weeks)
		2nd trimester (13–26 weeks)
		3rd trimester (27+ weeks)
Number of ANC visits	Number of antenatal care visits a pregnant woman made during her gestation period	4+
		3
		1–2

### Data management

Data extraction, cleaning, and analysis were done using Stata version 16 (Stata Corp, College Station, Tx, USA).

### Data analysis

Proportions and frequencies were used to summarize categorical variables in descriptive analysis. On the other hand, bivariate and multiple logistic regressions were used in analytical analysis.

Four stages of logistic regression modelling of survey data were applied as specified by Heeringa, West and Berglund [47]; and Hosmer, Lemeshow and Sturdivant [48]. First, bivariate analyses of the relationship of outcome to individual explanatory variable candidates were performed. Second, explanatory variables that had a bivariate association with the outcome at significance *p* < 0.25 were selected as candidates for the main effects in a multivariate logistic regression model. An initial model-building process using multivariate logistic regression analysis was done to further examine the association (measure of effect) between the outcome and each explanatory variable while controlling the effects of other explanatory variables. The model estimated adjusted odds ratios (AOR). The level of significance used was 5% (0.05), two-tailed at 95% confidence interval (CI). Third, the contribution of each explanatory variable to the multivariate model was evaluated using Wald test at 5% significant level (Table 2). Table 2 shows one of the six adjusted Wald tests is statistically significant and the predictor is “number of ANC visits”, in this initial model. This suggests that the parameters associated with the number of ANC visits in this logistic regression model are significantly different from zero and that the variable may be an important predictor of uptake of at least three doses of SP when adjusting for the relationships of the other predictor variables with the outcome. Therefore, at this stage in the model-building process, only the ‘number of ANC visits’ variable was retained of all of the candidates’ main effects. Thus, the second model-building process included the ‘number of ANC visits’ predictor only, which had similar odds ratios as the adjusted one. Lastly, scientifically justified interactions among the explanatory variables were also checked and there were no significant interactions observed.

**Table 2:** Design-Adjusted Wald tests for the parameters associated with categorical predictors in the initial multiple logistic regression model

Predictor	*F*-Test Statistic	*P*-value
Education level	*F*_(2,563)_ = 0.30	0.7399
Age group	*F*_(5,560)_ = 0.86	0.5088
Occupation	*F*_(2,563)_ = 0.14	0.8736
Wealth status	*F*_(4,561)_ = 0.92	0.4527
Parity	*F*_(4,561)_ = 0.37	0.8316
Timing of 1st ANC visit	*F*_(2,563)_ = 1.23	0.2925
Number of ANC visits	*F*_(2,563)_ = 25.01	<0.001*

* Design-adjusted Wald tests significant at the 0.05 level

The statistical analysis took into account the complex characteristics of the survey sample design by allowing adjustments for stratification, clustering and weighting for unequal selection probabilities.

### Ethical considerations

The Malawi Demographic and Health Survey protocol was reviewed and approved by Malawi’s National Health Sciences Research Committee and Inner City Fund (ICF) Institutional Review Board [46]. Interviewers informed prospective participants about the purpose of the study, procedures required of them if recruited, and that they had the right to volunteer whether or not to participate in the study [46]. Informed consent was obtained from each participant before administering the questionnaire and the respondents were assured of privacy and confidentiality [46]. To access the survey datasets, the author obtained permission from the Demographic and Health Surveys (DHS) Program. The datasets received were treated as confidential and no effort was made to identify any household or individual respondent interviewed in the survey. In addition, no ethical clearance was sought from Malawi’s National Health Sciences Research Committee because the research material collected for MDHS were not used differently in this study as stipulated in National Health Sciences Research Committee guidelines.

## Results

### Overall proportion of women who took three or more doses of IPTP-SP

Of the 1069 (weighted count) women, 447 (42, 95% CI: 38.1–45.6) received three (optimal) or more doses of IPTp-SP and 622 (58, 95% CI: 54.4–61.9) took less than three doses.

### Socio-demographic characteristics of participants and uptake of IPTp-SP

Out of 1069 women, 903 (84.5%) resided in rural areas and of these women, only 372 (41%) took at least three doses of IPTp-SP. Of 166 women who lived in urban areas, 45% took three or more doses of IPTp-SP during pregnancy. There is no statistically significant association between residential area and uptake of SP optimal doses (*p* = 0.5270). Almost two-thirds (65%) of participants had attended primary school and 12% had no formal education. Women who had attained secondary or higher education level had the highest uptake of three or more doses of IPTp-SP (49%) than the rest of women who achieved primary or no formal education levels. There is insignificant relationship between IPTp-SP uptake and education level (*p* = 0.1243). Participants in the age group 25–29 had the highest uptake of optimal SP doses compared to the rest of the age groups. Uptake of optimal dosages of SP varied significantly across age groups (*p* = 0.0318). More than half (51%) of employed women received at least three doses, the uptake of three or more dosages of SP was not statistically significant across occupation status (*p* = 0.1259). The richer and richest women had the highest uptake of optimal doses of SP than the rest. The uptake of the optimal doses of SP was not significantly different across the regions (*p* = 0.9412) (Table 3).

**Table 3:** Sociodemographic characteristics of participants by IPTp with SP uptake

Characteristic	N^†^ Total = 1069	% women took 3+ doses		Rao-Scott *F*-test	*p*-value
		n^‡^ (%)	(95%CI)		
Residence					
Urban	166	74 (45.0)	34.3–56.1	*F* (1, 564) = 0.4006	0.5270
Rural	903	372 (41.2)	37.3–45.2		
Education					
No formal education	125	48 (38.6)	29.1–49.1	*F* (2.00, 1126.79) = 2.0890	0.1243*
Primary	699	280 (40.0)	35.7–44.5		
Secondary or higher	245	119 (48.5)	40.7–56.3		
Age group					
15–19	168	66 (39.5)	31.4–48.2	*F* (4.85, 2736.04) = 2.4753	0.0318*
20–24	356	160 (45.1)	38.4–52.0		
25–29	237	117 (49.2)	40.9–57.5		
30–34	156	56 (36.0)	28.5–44.3		
35–39	108	34 (31.7)	22.6–42.5		
40+	44	13 (29.3)	17.2–45.2		
Occupation					
Unemployed	392	173 (44.2)	38.4–50.8	*F* (1.99, 1121.60) = 2.0785	0.1259*
Self-employed	578	224 (38.7)	33.9–43.9		
Salaried employment	99	50 (50.5)	40.3–63.2		
Wealth					
Poorest	234	96 (41.3)	33.6–49.4	*F* (3.90, 2196.87) = 1.8457	0.1193*
Poorer	239	89 (37.4)	30.2–45.1		
Middle	207	74 (35.7)	29.0–43.1		
Richer	199	96 (48.1)	39.7–56.6		
Richest	190	91 (48.0)	38.2–57.9		
Marital status					
Married	935	393 (42.0)	38.1–46.0	*F* (1.97, 1113.02) = 0.4090	0.6617
Divorced/separated/widowed	72	26 (36.4)	25.2–49.3		
Never married	62	28 (44.9)	30.5–60.2		
Region					
Southern	504	208 (41.3)	36.1–46.7	F (1.91, 1086.69) = 0.0302	0.9660
Central	447	188 (42.0)	36.0–48.2		
Northern	118	51 (43.2)	34.5–52.3		

^†^weighted total frequency, ^‡^weighted frequency of women who took three or more doses of IPTp-SP, *potential candidate for multivariate logistics regression because *p* < 0.25

### Obstetrical history and uptake of three or more doses of IPTp-SP

Table 4 displays the distribution of participants by obstetrical history that may affect the uptake of optimal IPTp-SP doses. Distribution of participants by reported number of children (parity); shows that mothers with five or more children had the lowest uptake of optimal doses of SP (32%) and the difference in SP uptake across parity was statistically significant (*p* = 0.0407). Study participants who started antenatal care in the first trimester had highest proportion of receiving optimal IPTp-SP (46%) compare to those who commenced in second and third trimesters (*p* = 0.0004). There was a significant relationship between the number of ANC visits and completion of recommended dosages of IPTp-SP (*p* < 0.001), with the highest uptake percent of 52% by those who made at least four visits to the ANC clinic.

**Table 4:** Association between obstetrical history and uptake of three or doses of IPTp with SP

Characteristic	N^†^ Total = 1069	% women took 3+ doses		Rao-Scott *F*-test	*p*-value
		n^‡^ (%)	(95%CI)		
Parity					
One child	277	125 (45.0)	37.4–52.9	*F* (3.83, 2158.02) = 2.5382	0.0407*
Two children	244	110 (45.0)	36.6–53.7		
Three children	161	79 (48.7)	40.1–57.3		
Four children	132	51 (38.6)	29.0–49.3		
Five or more children	254	82 (32.4)	26.8–38.6		
Timing of 1st ANC visit					
1st trimester	222	103 (46.2)	37.6–54.9	*F* (1.94, 1094.85) = 7.9535	0.0004*
2nd trimester	753	327 (43.5)	39.1–48.0		
3rd trimester	94	17 (17.8)	10.5–28.6		
Number of ANC visits					
4+	500	260 (52.0)	46.2–57.7	*F* (1.85, 1045.90) = 31.8155	<0.001*
3	413	172 (41.6)	35.7–47.7		
1–2	156	15 (9.9)	6.1–15.4		

^†^weighted total frequency, ^‡^weighted frequency of women who took three or more doses of IPTp-SP, *potential candidate for multivariate logistics regression because *p* < 0.25

In this study population, all women attended antenatal care at least once. Categorising the number of ANC visits as ‘1-2 only", ‘3 only’ and ‘4 or more’ the descriptive analysis results indicate that out of 1069 (weighted count) participants, 500 (46.8, 95% CI 43.4–50.2) attended ANC at least four times, 413 (38.6, 95% CI 35.2–42.1) made three ANC visits only and 156 (14.6, 95% CI 12.2–17.3) managed to attend ANC one-or two-times only during the entire gestation period. Cumulatively, the proportion of women that managed to attend at least three ANC clinics is 85.4% (95% CI 82.7–87.8).

**Fig. 2:**
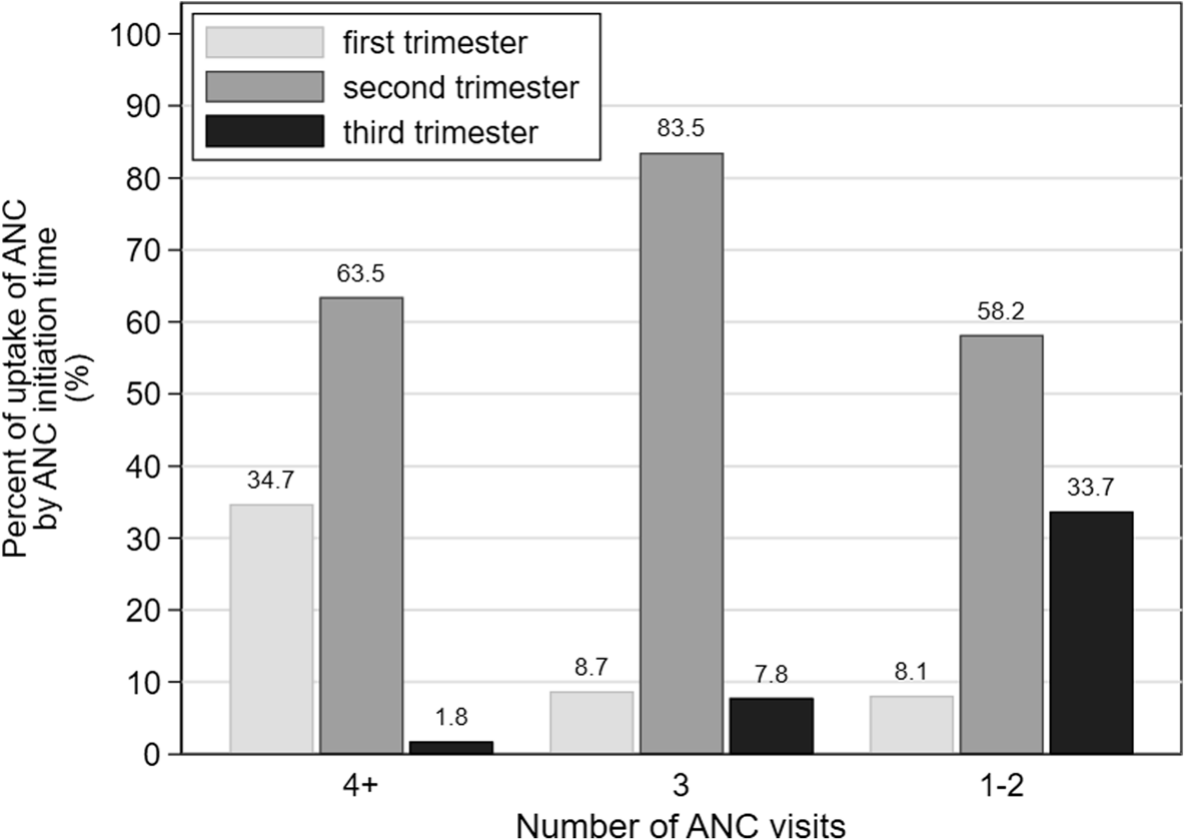
Proportion of uptake of antenatal care (ANC) against the number of ANC visits by ANC initiation time

Figure 2 indicates that almost two-thirds (64%) of the participants who made four or more ANC visits started antenatal care in second trimester while 35% initiated ANC in first trimester. Cumulatively 98% who made at least four ANC visits commenced ANC at the most in the second trimester. Majority (84%) who attended ANC thrice began their antenatal care in the second trimester, and cumulatively 92% started antenatal care in the second trimester at the most. Overall, 70.4% (95% CI 67.1–73.6) initiated ANC in the second trimester and 20.8% (95% CI 17.9–24.0) began antenatal care in the first trimester.

### Determinants of uptake of at least three doses of IPTp with SP

The results of multiple logistic regression analysis presented in Table 5 indicate that women who visited ANC clinics three times only during pregnancy have a marginally significant lower odds of completing the recommended number of IPTp-SP doses than those who made at least four ANC visits (*p* = 0.060) after controlling for the relationships of the other predictors. Furthermore, women who made one or two ANC visits only have significant lowest odds of receiving at least three IPTp with SP doses than those women who attended ANC four or more times, adjusting for the other explanatory variables in the model (*p* < 0.001).

**Table 5:** Estimates of Adjusted Odds ratios for the uptake of three or more doses of IPTp-SP

Characteristics	*N*^†^ = 1069 n^‡^	Adjusted Odds Ratio (95%CI)	*P*-value
Education^*a*^			
No formal education	125	1	
Primary	699	0.85 (0.52–1.39)	0.514
Secondary or higher	245	0.95 (0.51–1.75)	0.860
Age group^*a*^			
15–19	168	1	
20–24	356	1.37 (0.81–2.34)	0.241
25–29	237	1.67 (0.81–3.45)	0.166
30–34	156	1.17 (0.51–2.69)	0.708
35–39	108	0.97 (0.39–2.44)	0.956
40+	44	0.99 (0.35–2.87)	0.993
Occupation^*a*^			
Unemployed	392	1	
Self-employed	578	0.94 (0.66–1.33)	0.730
Employed	99	1.07 (0.60–1.93)	0.813
Wealth^*a*^			
Poorest	234	1	
Poorer	238	0.76 (0.47–1.22)	0.260
Middle	207	0.73 (0.46–1.18)	0.197
Richer	199	1.06 (0.64–1.73)	0.829
Richest	190	0.92 (0.51–1.67)	0.784
Parity^*a*^			
One child	277	1	
Two children	244	0.99 (0.57–1.77)	0.998
Three children	161	1.07 (0.55–2.06)	0.842
Four children	132	0.85 (0.36–1.99)	0.711
Five or more children	254	0.73 (0.32–1.66)	0.452
Timing of 1st ANC visit*			
2nd trimester	753	1	
1st trimester	222	0.85 (0.56–1.29)	0.453
3rd trimester	94	0.60 (0.29–1.23)	0.164
Number of ANC visits*			
4+	500	1	
3	413	0.71 (0.49–1.02)	0.060
1–2	156	0.12 (0.06–0.21)	<0.001

^*a*^Initial multiple logistic regression; ^†^weighted total frequency, ^‡^weighted frequency for each categorical variable, *second multiple logistic regression

## Discussion

The aims of the study are to estimate the proportion of and identify factors associated with the uptake of at least three doses of IPTp with SP among pregnant women in Malawi after adopting the updated WHO IPTp policy. The study findings have shown that overall proportion of women who took three or more doses of IPTp with SP is very low (42%) in the study population when compared to Roll Back Malaria (RBM) benchmark target of at least 80% for all pregnant women residing in areas with moderate-to-high malaria transmission in Africa [6]. A study from Uganda conducted in 2016 shows that uptake of three or more doses of IPTp-SP is 18% [49]. Studies reported from Ghana indicate that coverage of IPTp3+ is 46.6% in Tema metropolis of the Greater Accra region [39], 31.8% in Kumasi [50] and in Osu Klottey sub-district of Accra Metropolitan is 73.8% [51]. A study conducted in Chókwè district, southern Mozambique showed that coverage of three or more doses of SP is 46.6% [52]. It should be noted that Uganda, Ghana, and Mozambique adopted the updated WHO IPTp policy in 2014, 2012 and 2014 respectively [53]. Thus, the coverage of IPTp3+ in Malawi is reasonably similar to the aforementioned SSA countries post-adoption of 2012 WHO IPTp policy. The study reported by Henry et al. observed that majority of African countries have adopted the updated WHO IPTp-SP policy of providing three or more doses of IPTp to pregnant women but they are slow in fully implementing it [53], hence coverage estimates remain far below global targets [53, 54].

Among the nine covariates that were analysed to explain the uptake of at least three doses of IPTp with SP among pregnant women in Malawi, only the “number of ANC visits” variable was found to affect the uptake of SP after controlling for the other predictors.

The descriptive data analysis results of this study revealed that the proportion of receipt of three or more doses of IPTp with SP was highest among pregnant women making four or more ANC visits than those making fewer visits. Moreover, the adjusted odds ratio results indicate that pregnant women who attended ANC three times only and those who visited ANC one- or two-times only during gestation period had lower odds of receiving optimal doses of IPTp-SP compared to pregnant women who had made four or more ANC visits. This finding is consistent with studies done in Malawi [30, 31], Uganda [49], Tanzania [22, 33], Ghana [36, 39], Burkina Faso [34], Mali [35, 37], Cameroon, and in Benin [32].

Antenatal care clinic offers a platform for critical healthcare services and interventions such as health promotion, prevention, screening and diagnosis of diseases; aimed at improving maternal and foetal health [55, 56]. IPTp with SP is one of the preventive interventions being implemented at ANC clinics in Malawi. The 2012 updated WHO IPTp-SP policy recommends three or more doses of SP and that first dose should be administered as early as possible during the second trimester of gestation [21]. This means that early ANC initiation would increase the likelihood of receiving optimal SP doses as more visits would be made [21]. In this study majority initiated ANC after the first trimester but WHO recommends that pregnant women initiate ANC during the first trimester of pregnancy [29, 56]. The study results show that less than half of the participants attended ANC clinics at least four times, suggesting low uptake of Focused antenatal care (FANC) model that was used at the time of the study as a standard. FANC model sets a minimum of four antenatal care visits and assessments by or under the supervision of a skilled attendant during an uncomplicated pregnancy [29, 56]. The updated WHO IPTp policy was framed to be congruent with the Focused antenatal care (FANC) model in order to increase uptake of optimal doses of IPTp with SP [21, 29]. Worse still, only 52% of those women who made four or more ANC visits received the optimal doses of IPTp-SP. Moreover, 98 % of the women who attended ANC at least four times initiated the ANC clinic in the second trimester at the most. This suggests a missed opportunity to provide the recommended doses of IPTp with SP to women who managed at least four ANC visits [20].

The discrepancy between percentage of four or more ANC visits and percentage of receipt of at least three doses of SP could hs partly because of intermittent shortage of SP in the health facilities [38], poor fidelity in implementation of IPTp-SP intervention by individual healthcare providers as recommended in WHO policy brief that includes pregnant women taking IPTp-SP doses under Directly Observed Therapy (DOT) [57], and women’s negative attitudes towards the use of the drug during pregnancy [5, 22, 35, 58]. Another explanation could be that some of the ANC visits were not scheduled because of unexpected complications, hence SP doses were not dispensed.

The use of a nationally representative sample could strengthen the generalizability of the results to Malawian women with live births on uptake of optimal doses of IPTp with SP and its associated factors. However, the study had some limitations. First, the analysis was limited on the variables that were available in the MDHS questionnaire associated with IPTp uptake; thus, the author could not explore other factors that could be relevant for this study, for example, beliefs/perception/attitude of the participants and their partners towards IPTp. Second, the study did not address issues of stockpiling and the availability of SP doses at the health facilities. There is a possibility that women who attended the WHO-recommended minimum number of ANC clinics failed to receive prophylaxis because the commodity was not available. Third, the study excluded women who had a stillbirth and those whose children died before birth. The exclusion may have created selection bias that may have under-or overestimate the SP uptake if the excluded population differs from those included in the study in regard to SP uptake, ANC attendance, the timing of the first ANC visit, and any other explanatory variables. In this regard, the findings of this study are only generalisable to women with live children at birth. Finally, one of the survey design inherent limitation is recall bias. Women may have reported past exposures/experiences with varying degree of accuracy because the primary source of the information collected from the research participants was self-reported. This may result in underestimation or overestimation of past experiences or events. However, some of these recall biases were minimised by interviewing the women less than 2 years after delivering live children.

## Conclusion

The results demonstrate that there is low uptake of the WHO-recommended three or more SP doses and this seems to be associated with the number of ANC visits. Moreover, the four or more ANC visits have limited effectiveness on the uptake of optimal SP doses in this study population. Thus, there is a need for continued and varied efforts to increase both the uptake of optimal doses of IPTp with SP, WHO-recommended ANC number of contacts (visits) and effectiveness of ANC services. Future studies should explore health facility-based factors that could influence IPTp uptake, such as accessibility of drugs at clinics (that includes stock levels of SP), skills and knowledge of ANC providers, pregnant women taking SP doses under Directly Observed Therapy (DOT) and if there is proper documentation of the SP uptake in women’s ANC cards and any appropriate health facility ANC registers.

## Data Availability

The study used data from the Demographic and Health Surveys (DHS) Program with permission. The data are publicly available and may be requested from the DHS Program office on (https://www.dhsprogram.com/data/dataset_admin). The questionnaire used for the analyses is the women’s questionnaire contained within the MDHS, which can be accessed publicly at https://www.dhsprogram.com/publications/publication-fr319-dhs-final-reports.cfm?cssearch=12978_1.

## Abbreviations

ANC: Antenatal Care
FANC: Focused antenatal care
IPTp3 +: Three or more doses of IPTp-SP.
IPTp-SP: Intermittent Preventive Treatment in pregnancy with Sulphadoxine Pyrimethamine
MiP: Malaria in pregnancy
SP: Sulphadoxine Pyrimethamine
WHO: World Health Organization

## References

[1] Dellicour S, Tatem A, Guerra C, Snow R, ter Kuile F (2010). Quantifying the number of pregnancies at risk of malaria in 2007: a demographic study. PLoS Med.

[2] Guyatt H, Snow R (2001). The epidemiology and burden of plasmodium falciparum -related anemia among pregnant women in sub-Saharan Africa. Am J Trop Med Hyg.

[3] Menéndez C, D'Alessandro U, ter Kuile FO (2007). Reducing the burden of malaria in pregnancy by preventive strategies. Lancet Infect Dis.

[4] Desai M, ter Kuile F, Nosten F, McGready R, Asamoa K, Brabin B, Newman R (2007). Epidemiology and burden of malaria in pregnancy. Lancet Infect Dis.

[5] Hill J, Hoyt J, van Eijk AM, D'Mello-Guyett L, Ter Kuile FO, Steketee R, Smith H, Webster J (2013). Factors affecting the delivery, access, and use of interventions to prevent malaria in pregnancy in sub-Saharan Africa: a systematic review and meta-analysis. PLoS Med.

[6] Partnership RBM. The contribution of malaria control to maternal and newborn health. In: Progress & impact Geneva: World Health Organization; 2014. https://apps.who.int/iris/bitstream/handle/10665/126340/9789241507219_eng.pdf.

[7] Agomo CO, Oyibo WA, Odukoya-Maije F (2011). Parasitologic assessment of two-dose and monthly intermittent preventive treatment of malaria during pregnancy with Sulphadoxine-Pyrimethamine (IPTP-SP) in Lagos. Nigeria Malar Res Treat.

[8] Malaria and Pregnancy [http://www.malariasite.com/pregnancy/].

[9] Dimasuay KG, Aitken EH, Rosario F, Njie M, Glazier J, Rogerson SJ, Fowkes FJ, Beeson JG, Powell T, Jansson T (2017). Inhibition of placental mTOR signaling provides a link between placental malaria and reduced birthweight. BMC Med.

[10] Rogerson SJ, Mwapasa V, Meshnick SR (2007). Malaria in pregnancy: linking immunity and pathogenesis to prevention. Am J Trop Med Hyg.

[11] Steketee R, Nahlen B, Parise M, Menendez C (2001). The burden of malaria in pregnancy in malaria-endemic areas. Am J Trop Med hyg.

[12] Lagerberg RE (2008). Malaria in pregnancy: a literature review. J Midwifery Womens Health.

[13] Kayentao K, Garner P, van Eijk AM, Naidoo I, Roper C, Mulokozi A, MacArthur JR, Luntamo M, Ashorn P, Doumbo OK (2013). Intermittent preventive therapy for malaria during pregnancy using 2 vs 3 or more doses of sulfadoxine-pyrimethamine and risk of low birth weight in Africa: systematic review and meta-analysis. Jama.

[14] Eisele TP, Larsen DA, Anglewicz PA, Keating J, Yukich J, Bennett A, Hutchinson P, Steketee RW (2012). Malaria prevention in pregnancy, birthweight, and neonatal mortality: a meta-analysis of 32 national cross-sectional datasets in Africa. Lancet Infect Dis.

[15] Guyatt H, Snow R (2004). Impact of malaria during pregnancy on low birth weight in sub-Saharan Africa. Clin Microbiol Rev.

[16] National Malaria Control Programme (NMCP) [Malawi] (2010). Malawi Strategic Plan 2011–2015: Towards Universal Acess.

[17] Feng G, Simpson JA, Chaluluka E, Molyneux ME, Rogerson SJ (2010). Decreasing burden of malaria in pregnancy in Malawian women and its relationship to use of intermittent preventive therapy or bed nets. PLoS One.

[18] WHO. A Strategic Framework for Malaria Prevention and Control During Pregnancy in the African Region. In:., vol. (AFR/MAL/04/01). Brazzaville, Congo: World Health Organization Regional Office for Africa;2004. http://whqlibdoc.who.int/afro/2004/AFR_MAL_04.01.pdf.

[19] WHO. Malaria in Pregnancy Guidelines for Measuring Key Monitoring and Evaluation Indictors. 2007. http://whqlibdoc.who.int/publications/2007/9789241595636_eng.pdf. Accessed 24 Nov 2015.

[20] Andrews KG, Lynch M, Eckert E, Gutman J (2015). Missed opportunities to deliver intermittent preventive treatment for malaria to pregnant women 2003-2013: a systematic analysis of 58 household surveys in sub-Saharan Africa. Malar J.

[21] WHO (2012). Updated WHO policy recommendation: intermittent preventive treatment of malaria in pregnancy using sulfadoxine-pyrimethamine (IPTp-SP).

[22] Mpogoro FJ, Matovelo D, Dosani A, Ngallaba S, Mugono M, Mazigo HD (2014). Uptake of intermittent preventive treatment with sulphadoxine-pyrimethamine for malaria during pregnancy and pregnancy outcomes: a cross-sectional study in Geita district, North-Western Tanzania. Malar J.

[23] ter Kuile FO, van Eijk AM, Filler SJ (2007). Effect of sulfadoxine-pyrimethamine resistance on the efficacy of intermittent preventive therapy for malaria control during pregnancy: a systematic review. Jama.

[24] ter Kuile FO, Steketee RW (2007). Intermittent preventive therapy with sulfadoxine-pyrimethamine during pregnancy: seeking information on optimal dosing frequency. J Infect Dis.

[25] WHO (2012). Evidence review group: Intermittent preventive treatment of malaria in pregnancy (IPTp) with sulfadoxine–pyrimethamine (SP).

[26] Desai M, Hill J, Fernandes S, Walker P, Pell C, Gutman J, Kayentao K, Gonzalez R, Webster J, Greenwood B. Prevention of malaria in pregnancy. Lancet Infect Dis. 2018;18(4):e119–32. 10.1016/S1473-3099(18)30064-1 29395997

[27] Mwendera CA, Jager C, Longwe H, Phiri K, Hongoro C, Mutero CM (2017). Changing the policy for intermittent preventive treatment with sulfadoxine–pyrimethamine during pregnancy in Malawi. Malar J.

[28] Malawi National Malaria Control Programme (2013). Revised guidelines for the treatment of malaria in Malawi.

[29] WHO (2006). Provision of effective antenatal care, Integrated management of pregnancy and childbirth (IMPAC). Standards for maternal and neonatal care.

[30] Azizi SC, Chongwe G, Chipukuma H, Jacobs C, Zgambo J, Michelo C (2018). Uptake of intermittent preventive treatment for malaria during pregnancy with Sulphadoxine-Pyrimethamine (IPTp-SP) among postpartum women in Zomba District, Malawi: a cross-sectional study. BMC Pregnancy Childbirth.

[31] Nkoka O, Chuang T-W, Chen Y-H (2018). Association between timing and number of antenatal care visits on uptake of intermittent preventive treatment for malaria during pregnancy among Malawian women. Malar J.

[32] d'Almeida TC, Agboton-Zoumenou MA, Garcia A, Massougbodji A, Briand V, Imorou Y, Cottrell G (2011). Field evaluation of the intermittent preventive treatment of malaria during pregnancy (IPTp) in Benin: evolution of the coverage rate since its implementation. Parasit Vectors.

[33] Exavery A, Mbaruku G, Mbuyita S, Makemba A, Kinyonge IP, Kweka H (2014). Factors affecting uptake of optimal doses of sulphadoxine-pyrimethamine for intermittent preventive treatment of malaria in pregnancy in six districts of Tanzania. Malar J.

[34] Gies S, Coulibaly SO, Ky C, Ouattara FT, Brabin BJ, D'Alessandro U (2009). Community-based promotional campaign to improve uptake of intermittent preventive antimalarial treatment in pregnancy in Burkina Faso. Am J Tropical Med Hyg.

[35] Hill J, Kayentao K, Touré M, Diarwara S, Bruce J, Smedley J, Doumbo OK, Kuile FO, Webster J (2014). Effectiveness of antenatal clinics to deliver intermittent preventive treatment and insecticide treated nets for the control of malaria in pregnancy in Mali: a household survey. PLoS One.

[36] Hommerich L, von Oertzen C, Bedu-Addo G, Holmberg V, Acquah PA, Eggelte TA, Bienzle U, Mockenhaupt FP (2007). Decline of placental malaria in southern Ghana after the implementation of intermittent preventive treatment in pregnancy. Malar J.

[37] Leonard N, Eric FB, Judith AK, Samuel W (2016). Factors associated to the use of insecticide treated nets and intermittent preventive treatment for malaria control during pregnancy in Cameroon. Arch Public Health.

[38] Pell C, Straus L, Andrew EVW, Meñaca A, Pool R (2011). Social and cultural factors affecting uptake of interventions for malaria in pregnancy in Africa: a systematic review of the qualitative research. PLoS One.

[39] Amankwah S, Anto F (2019). Factors associated with uptake of intermittent preventive treatment of malaria in pregnancy: a cross-sectional study in private health facilities in Tema Metropolis, Ghana. J Trop Med.

[40] Mubyazi GM, Bygbjerg IC, Magnussen P, Olsen O, Byskov J, Hansen KS, Bloch P (2008). Prospects, achievements, challenges and opportunities for scaling-up malaria chemoprevention in pregnancy in Tanzania: the perspective of national level officers. Malar J.

[41] World Health Organization (2013). Factsheet on the World Malaria Report.

[42] Kibusi SM, Kimunai E, Hines CS (2015). Predictors for uptake of intermittent preventive treatment of malaria in pregnancy (IPTp) in Tanzania. BMC Public Health.

[43] Mwandama D, Gutman J, Wolkon A, Luka M, Jafali J, Ali D, Mathanga DP, Skarbinski J (2015). The use of intermittent preventive treatment in pregnancy and insecticide-treated bed nets for malaria prevention by women of child-bearing age in eight districts in Malawi. Malar J.

[44] Launiala A, Honkasalo M-L (2007). Ethnographic study of factors influencing compliance to intermittent preventive treatment of malaria during pregnancy among Yao women in rural Malawi. Trans R Soc Trop Med Hyg.

[45] Ayubu MB, Kidima WB (2017). Monitoring compliance and acceptability of intermittent preventive treatment of malaria using sulfadoxine pyrimethamine after ten years of implementation in Tanzania. Malaria Res Treat.

[46] National Statistical Office/Malawi (2017). ICF: Malawi Demographic and Health Survey 2015–16.

[47] Heeringa SG, West BT, Berglund PA (2017). Applied survey data analysis: chapman and hall/CRC.

[48] Hosmer DW Jr, Lemeshow S, Sturdivant RX. Applied logistic regression, vol. 398: Wiley; 2013. https://apps.who.int/iris/bitstream/handle/10665/126340/9789241507219_eng.pdf.

[49] Okethwangu D, Opigo J, Atugonza S, Kizza CT, Nabatanzi M, Biribawa C, Kyabayinze D, Ario AR (2019). Factors associated with uptake of optimal doses of intermittent preventive treatment for malaria among pregnant women in Uganda: analysis of data from the Uganda demographic and health survey, 2016. Malar J.

[50] Addai-Mensah O, Annani-Akollor ME, Fondjo LA, Sarbeng K, Anto EO, Owiredu E-W, Arthur SN (2018). Regular antenatal attendance and education influence the uptake of intermittent preventive treatment of malaria in pregnancy: a cross-sectional study at the university hospital, Kumasi, Ghana. J Trop Med.

[51] Owusu-Boateng I, Anto F (2017). Intermittent preventive treatment of malaria in pregnancy: a cross-sectional survey to assess uptake of the new sulfadoxine–pyrimethamine five dose policy in Ghana. Malar J.

[52] Arnaldo P, Rovira-Vallbona E, Langa JS, Salvador C, Guetens P, Chiheb D, Xavier B, Kestens L, Enosse SM, Rosanas-Urgell A (2018). Uptake of intermittent preventive treatment and pregnancy outcomes: health facilities and community surveys in Chókwè district, southern Mozambique. Malar J.

[53] Henry M, Florey L, Youll S (2018). Gutman JRJMj: an analysis of country adoption and implementation of the 2012 WHO recommendations for intermittent preventive treatment for pregnant women in sub-Saharan Africa. Malar J.

[54] Agarwal K, Alonso P, Chico RM, Coleman J, Dellicour S, Hill J, Majeres-Lugand M, Mangiaterra V, Menendez C, Mitchell K (2015). Global call to action to scale-up coverage of intermittent preventive treatment of malaria in pregnancy: seminar report. Malar J.

[55] Tunçalp Ӧ, Pena-Rosas JP, Lawrie T, Bucagu M, Oladapo OT, Portela A, Gülmezoglu AM (2017). WHO recommendations on antenatal care for a positive pregnancy experience-going beyond survival. J BJOG.

[56] Pell C, Meñaca A, Were F, Afrah NA, Chatio S, Manda-Taylor L, Hamel MJ, Hodgson A, Tagbor H, Kalilani L (2013). Factors affecting antenatal care attendance: results from qualitative studies in Ghana. Kenya and Malawi PloS one.

[57] WHO (2013). World Health Organization, WHO Policy Brief for the Implementation of Intermittent Preventive Treatment of Malaria in Pregnancy using Sulfadoxine-Pyrimethamine (IPTp-SP).

[58] Bausell L, Wolf K (2015). Treatment uptake and availability of antimalarial drugs for intermittent preventative treatment in pregnant women in Malawi.

